# Level, causes, and risk factors of stillbirth: a population-based case control study from Chandigarh, India

**DOI:** 10.1186/s12884-017-1557-4

**Published:** 2017-11-13

**Authors:** Ariarathinam Newtonraj, Manmeet Kaur, Madhu Gupta, Rajesh Kumar

**Affiliations:** Department of Community Medicine, School of Public Health, Post Graduate Institute of Medical Education and Research, Chandigarh, 160 012 India

**Keywords:** Stillbirth, Fetal death, Case control, Capture and recapture, Risk factors, India, Pregnancy outcome, Incidence

## Abstract

**Background:**

Globally, India ranks first in the absolute number of stillbirths. Hence, the level, causes, and risk factors of stillbirths were estimated to facilitate designing of prevention strategy.

**Methods:**

Capture and recapture method was used to identify 301 stillbirths from 1st July 2013 to 31st August 2014 in Chandigarh Union Territory of India. Verbal autopsies (*n* = 181) were done at household level to identify causes of stillbirths. Risk factors were determined using case-control approach. Women who had a stillbirth in the past 3 months were enrolled as cases (*n* = 181) and those who had live-birth in same neighbourhood were included as controls (n = 181). Statistical differences in the distribution of characteristics of cases and controls were tested by t test and chi square test respectively for quantitative and categorical variables. In logistic regression models adjusted odds ratios (aOR) and 95% confidence intervals (CIs) were estimated for various risk factors.

**Results:**

Stillbirth rate was estimated to be 16/1000 birth. Antepartum causes were more common (68%) than intrapartum causes (32%). Among maternal conditions, hypertension (18.2%) and chorio-amnionitis (13.8%), and among foetal conditions, growth restriction (19.9%) and congenital anomalies (18.8%) were the leading causes. In about half of the stillbirths foetal (48%) and maternal (44.7%) causes were unidentifiable. Risk factors of stillbirths were: higher maternal age (aOR 1.1, 95%CI 1.0–1.2), vaginal delivery (aOR 8.1, 95%CI 2.6–26), induced labour (aOR 2.6, 95%CI 1.5–4.5), green or light brown liquor (aOR 2.0, 95%CI 1.1–3.8), preterm delivery (aOR 6.4, 95%CI 3.7–11) and smaller household size (aOR 1.2, 95% CI 1.1–1.3).

**Conclusions:**

Stillbirth rate was high in Chandigarh Union Territory of India. Major causes and risk factors amenable to interventions were infections, hypertension, congenital malformations, foetal growth restriction, pre-maturity and household size. Therefore, better maternity ante-natal and intra-natal care is required to achieve a single digit stillbirth rate.

**Electronic supplementary material:**

The online version of this article (10.1186/s12884-017-1557-4) contains supplementary material, which is available to authorized users.

## Background

Stillbirths constitute a major part of perinatal deaths, yet they largely remain invisible [1]. Worldwide about 2.65 million babies were born as stillbirth in 2008. Out of these about 98% of the third-trimester stillbirths occurred in low-income and middle-income countries. However, most of the high quality epidemiological studies have been conducted in high income countries [1], leading to a worldwide 10/90 gap in health research; only 10% of the research addresses 90% of the burden [2]. This gap is wider in low income countries, where very few studies have been conducted [3]. Hence, better information on the extent of stillbirths, their causes and risk factors is needed from low- and middle-income countries for planning prevention programmes [1, 2, 4].

Globally, India has been ranked first in the absolute number of stillbirths [4, 5]. However, the sample registration system (SRS) of India has estimated stillbirth rate to be only 5 per 1000 births in 2013 [6]. Whereas, Blencowe et al. (2016) have estimated it to be 23 per 1000 live births [5, 6]. Wide range of variation in stillbirth rate (12.5 to 26.48) has been reported across the states of India [7–11]. Recently, Government of India has set a target for bringing down the stillbirth rate to single digit by 2025 [12].

In response to the commitment to the 67th World Health Assembly held in May 2014, New-born Action Plan (INAP) has been launched in India to end preventable newborn deaths and stillbirths by 2030 [13, 14]. Therefore, a population-based stillbirth surveillance systems is required to track this indicator in all states. Consequently, a stillbirth review system has been started in Chandigarh Union Territory of India where nearly two third of the women do not receive full antenatal care [15]. Aim of this study was to estimate stillbirth rate and to determine the causes and risk factors of stillbirths so as to facilitate designing of a stillbirth prevention strategy.

## Methods

### Study setting

This study was conducted in Chandigarh, a Union Territory (UT), located in northern part of India, which was having a population of 1,055,450 in 2011 [16]. About 97% the population reside in urban area and 3% live in rural area [17].

### Study design

This study used following three approaches. Firstly, to estimate stillbirth rate, ‘catch and re-catch’ method was used [9]. Secondly, to assess probable causes of stillbirth verbal autopsy technique was utilized followed by coding of the causes according to the International Classification of Diseases, Tenth Revision (ICD-10) and classification of causes according to the schema proposed by Lawn et al. (2011) [4, 18, 19]. Thirdly, to determine the risk factors of stillbirths, a case-control design was adopted.

### Study participants

**Fig. 1:**
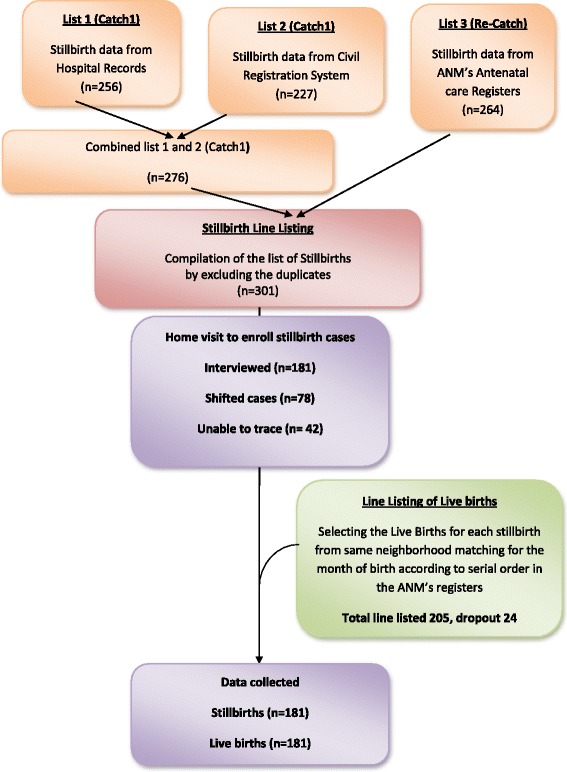
Selection procedures for the study participants

All stillbirths from 1st July 2013 to 31st August 2014 were registered prospectively from multiple agencies (hospitals, community health workers, and birth registration office). Cases were women (18–45 years) who had stillbirths in the past 3 months from the date of interview and the controls were mothers having live-birth, in the same time frame, residing in the same area as that of case (Fig. 1).

### Study tools

Data were collected using three questionnaires. First questionnaire was on verbal autopsy which was adapted from World Health Organization (WHO) verbal autopsy standard manual [18]. The second questionnaire was prepared on the basis of review of literature to capture additional information from hospital records. And the third questionnaire was on Social Capital which had been developed by Narayan et al. (2001) and it was earlier used by World Bank [18, 20–23]. These questionnaires were pretested and translated in local language before use.

### Data collection

Four interviewers having graduate level qualifications were recruited and trained. The data collection was done in two steps. First step was to identify all stillbirths among residents of Chandigarh Union Territory (UT) from the records, and the second step involved locating the women in the community who had delivered a stillborn baby (case) and selection of women who had a live-birth (control) in the same neighbourhood as that of the case (Fig. 1).

To identify all stillbirths in Chandigarh UT, the data were collected from three sources: (a) five major hospitals (b) office of the registrar of birth and death known as Civil Registration System, and (c) community-based auxiliary nurse midwives (ANMs). After collecting data from above three sources, duplications were removed, and final list of stillbirths was prepared.

One control (live-birth) for each case (stillbirth) was selected systematically from the ANM’s Birth Register from the same neighbourhood area to which the case belonged, matching for the month of birth also. The controls were selected according to the serial number of the register to avoid selection bias i.e., first live-birth in the same month when stillbirth had occurred. After the selection of cases (stillbirths) and controls (live-births), the interviewers visited the households to interview the respondents.

A total of 301 stillbirths were line-listed. Out of these, 181 mothers could be contacted; rest of the mothers were found to be either shifted out of the city after the birth of the baby (*n* = 78) or could not be found at the address which was provided to the hospital (*n* = 42). A total of 205 live births (controls) were enrolled but 181 (88.3%) mothers could be interviewed. Twenty four mothers could not be interviewed due to their non-availability despite repeated visit (*n* = 19) and due to refusal to give consent (*n* = 5).

### Statistical analysis

Following variable definitions were used in the study:

*Stillbirth:* As per World Health Organization (WHO) definition, operationally, stillbirth was defined as ‘a late foetal death with ≥ 28 completed weeks of gestation’. Recent estimates of stillbirths published in The Lancet were based on the same operational definition [4, 5, 24]. The weight and body length were not taken into consideration to define stillbirth as it was not feasible to do so.

*Antepartum and Intrapartum stillbirth:* Classification of antepartum stillbirth and intrapartum stillbirth was done mainly based on history of ‘baby stopped moving’ and or ‘baby looked macerated’. In addition, responses to two open-ended questions were also taken into consideration, which were asked at the end of interview to ascertain the cause of stillbirth: (a) what did doctor tell were the causes of the death of this baby, and (b) in your opinion when did the baby die- before labour or during labour?

*Socio-economic status:* It was assessed using modified Kuppuswamy scale 2014 [25].

*Social capital:* It captures the existence of community networks; civic engagement; local identity and a sense of solidarity and equity with other community members; and trust and reciprocal help and support [26]. World Bank report in 2004 added few more dimensions, i.e., groups and networks; trust and solidarity; collective action and cooperation; information and communication; social cohesion and inclusion; empowerment and political action [23].

*Maternal hypertension:* Mothers was diagnosed with hypertension before or during the pregnancy, or having pre-eclampsia or eclampsia during the pregnancy were included in this category [27].

*Foetal growth:* Small-for-gestational-age (SGA), large-for-gestational-age (LGA) and appropriate-for-gestational-age (AGA) were classified using Fenton’s chart wherever sex and weight were available from the health record [28].

*Gestational age:* It was estimated on the basis of the reported date of last menstrual period.

*Preterm:* A baby born less than 37 weeks of gestation was considered to be pre-term.

*Congenital malformation:* Following six verbal autopsy questions were used to identify congenital malformations; whether the head was not formed or smaller head, whether the head of the child was larger, any swelling or opening in the back of the baby, whether there was any defect in the palate, any deformity in the hand and feet or any other malformation.

#### Estimation of stillbirth rate

The number of live-births in the population of Chandigarh in 2014 were estimated by using the crude birth rate reported by the Sample Registration System (SRS) in 2014 and by projecting the population from 2011 census to year 2014 using the population growth rate reported by Census of India for Chandigarh. The number of stillbirths were estimated using capture-recapture method.

In the capture and recapture, Lincoln-Petersen unbiased formula (N) = [(A + 1) × (B + 1)/(AB + 1)] − 1 was used, wherein ‘A’ (*n* = 276) denotes the number of stillbirths captured from the hospital records and Civil Registration System (CRS), ‘B’ (*n* = 264) denotes the number of stillbirths captured from the community-based records of Auxiliary Nurse Midwife (ANM), and ‘AB’ (*n* = 239) denotes the number of stillbirths captured by both the system [9]. We have clubbed Civil Registration System (CRS) and Hospital data as one catch, because Hospitals report to Civil Registration System (CRS), hence, these cannot be considered as independent sources. Assumption of capture and recapture method is that sources should be independent and the chance of being captured by each source should be equal [9]. The clubbing of data helped us to fill some missing data from one of the tertiary care hospital which had refused to share data with us. Stillbirth rate was defined as number of stillbirth per 1000 births in one calendar year.

#### Causes of stillbirths

Stillbirths were classified according to the method described by Lawn et al. (2011), where they classified the stillbirth into intrapartum and antepartum, and most likely maternal cause and foetal cause [4]. The verbal autopsies and available health records of 181 stillbirths were reviewed by two community physicians separately. They assigned one foetal and one maternal cause code according to the International Classification of Diseases (ICD) 10th revision [4, 19]. If there was a consensus code between two community physicians, then that code was taken as a final cause. If there was no consensus between the two community physicians, a third community physician of a higher rank reviewed the case and assigned a final cause.

#### Risk factors of stillbirths

Statistical differences in the characteristics of cases and controls were compared by t-test for quantitative variables and by Pearson’s chi square test or fisher’s exact test for categorical variables. Logistic regression models were fitted to adjust for the effects of confounding variables. All variables were considered while arriving at the final model to evaluate the risk of stillbirth by estimation of adjusted odds ratio (aOR) with 95% confidence intervals (CI). Data were analysed using SPSS 22.0.0 (Statistical Package for the Social Sciences).

## Results

### Stillbirth rate

During the study period, 301 stillbirths were recorded in Chandigarh UT. The stillbirth rate was estimated to be 16 per 1000 births per year. Use of capture and recapture method also predicted the same rate.

### Causes of stillbirths

On the basis of verbal autopsy and available health records, 124 (68%) stillbirths were classified as antepartum and 57 (32%) as intra-partum (Table 1). Antepartum and intra-partum causes were classified further into foetal and associated maternal conditions. In antepartum causes, the foetal causes were ‘unidentifiable’ in 46%. Intra-Uterine Growth Retardation (IUGR) was the commonest cause (19.4%) followed by congenital anomalies (18.5%). Among the associated maternal conditions, 49.2% had ‘unidentifiable’ conditions. Maternal hypertension accounted for 19.4% followed by chorio-amnionitis in 13.7%. Intra-partum foetal causes were ‘unidentifiable’ in 52.6%, Intra-Uterine Growth Retardation (IUGR) (21.1%) was noted as the commonest cause followed by congenital anomaly (19.3%). Among the associated maternal conditions, 35.0% had ‘unidentifiable’ conditions. Maternal hypertension, abnormal labour were more common (15.8% each) followed by chorio-amnionitis (14%). No case of uterine rupture was reported. Maternal and foetal causes were also cross-tabulated to find association of maternal and foetal causes (see Additional file 1).

**Table 1:** Medical causes of stillbirths in Chandigarh Union Territory, India, 2013–14

Cause	Antepartum stillbirths *N*=124 *n* (%)	Intrapartum stillbirths *N*=57 *n* (%)	Total *N*=181 *n* (%)
Foetal cause			
Congenital anomaly	23 (18.5)	11(19.3)	34 (18.8)
Infection or chorio-amnionitis	13 (10.5)	4 (7.0)	17 (9.4)
Foetal growth restriction/ placental insufficiency	24 (19.4)	12 (21.1)	36 (19.9)
Other specific foetal conditions	7 (5.6)	0 (0.0)	7 (3.9)
No conditions identified	57 (46.0)	30 (52.6)	87 (48.0)
Associated maternal condition			
Abnormal labour	0 (0.0)	9 (15.8)	9 (5.0)
Maternal hypertension	24 (19.4)	9 (15.8)	33 (18.2)
Maternal infection (e.g. Syphilis, Human Immuno-deficiency Virus)	1 (0.8)	0 (0.0)	1 (0.6)
Chorio-amnionitis	17 (13.7)	8 (14.0)	25 (13.8)
Maternal diabetes	2 (1.6)	1 (1.8)	3 (1.7)
Antepartum haemorrhage (abruption or placenta previa)	2 (1.6)	4 (7.0)	6 (3.3)
Maternal pre-existing condition (e.g., cardiac)^a^	2 (1.6)	1 (1.8)	3 (1.7)
Spontaneous preterm labour	13 (10.5)	3 (5.3)	16 (8.8)
Other maternal specific^b^	2 (1.6)	2 (3.5)	4 (2.2)
No maternal conditions identified	61 (49.2)	20 (35.0)	81(44.7)

^a^Two cases of cardiac disease and one case of beta-thalassemia. ^b^Three cases of hypothyroidism and one case of ABO incompatibility.

### Risk factors of stillbirths

The distribution of socio-economic status, place of residence, caste, and religion were not significantly different in cases and controls (data not shown). Statistically significant differences observed in the socio-demographic and medical factors of cases and control are presented in Table 2. Logistic regression model, shown in Table 3, revealed following risk factors of stillbirths: older age of mother (aOR 1.1, 95%CI 1.0–1.2), vaginal delivery (aOR 8.1, 95%CI 2.6–26), induced labour (aOR 2.6, 95%CI 1.5–4.5), green or light brown liquor (aOR 2.0, 95%CI 1.1–3.8), preterm delivery (6.4, 95%CI 3.7–11) and smaller number of household members (aOR 1.2, 95%CI 1.1–1.3).

**Table 2:** Distribution of socio-demographic and maternal factors among stillbirths and live-births

Characteristics	Stillbirths (cases) *N*=181	Live births (Controls) *N*=181	*p* value
Socio-demographic factors#	Mean (SD)	Mean (SD)	
Age of mother (years)	26.6 (4.4)	25.5 (3.7)	0.01
No. of household members	4.9 (2.5)	6.2 (2.9)	< 0.001
Social capital score	39.0 (7.8)	40.8 (8.0)	0.037
Maternal factors*	*n* (%)	*n* (%)	
Place of delivery			0.002
Home delivery/On the way to hospital	14 (5.5)	12 (5.0)	
Medical college (MC)	24 (13.3)	6 (3.3)	
Hospitals other than MC	143 (79.0)	163 (90.0)	
Mode of delivery			< 0.001
Caesarean section	6 (3.3)	35 (19.3)	
Vaginal	175 (96.7)	146 (80.7)	
Labour initiation			< 0.001
Spontaneous	81 (44.8)	105 (58.0)	
Induced	95 (52.4)	56 (31.0)	
Don’t know	5 (2.8)	20 (11.0)	
Colour of liquor			< 0.001
Clear	52 (28.7)	91 (50.3)	
Green or light brown	49 (27.1)	36 (19.9)	
Others/ Don’t know	80 (44.2)	54 (29.8)	
Sex of foetus			0.005
Male	76 (42.0)	103 (56.9)	
Female	103 (56.9)	78 (43.1)	
Don’t know	2 (1.1)	0	
Gestational age			< 0.001
≥ 37 weeks	70 (38.7)	147 (81.2)	
≤ 36 weeks	111 (61.3)	34 (18.8)	

*p* value determined by # t-test and * chi-square or fisher’s exact test

**Table 3:** Risk factors of stillbirth, Chandigarh Union Territory, India

Characteristics	Crude odds ratio (95% confidence intervals), *p*	Adjusted odds^a^ ratios (95% confidence intervals), *p*
Age of mother (years)	1.1 (1.0-1.1), 0.012	1.1 (1.0-1.2), 0.005
Smaller number of household members	1.2 (1.1-1.2), < 0.001	1.2 (1.1-1.3), < 0.001
Mode of delivery		
Caesarean section	1	1
Vaginal	7.0 (2.9-17.1), < 0.001	8.1 (2.6-26), < 0.001
Labour initiation		
Spontaneous	1	1
Induced	2.2 (1.4-3.4), < 0.001	2.6 (1.5-4.5), 0.001
Colour of liquor		
Clear	1	1
Green or light brown	2.4 (1.4-4.1), 0.002	2.0 (1.1-3.8), 0.03
Gestational age		
≥ 37 weeks	1	1
≤ 36 weeks	6.9 (4.3-11.1), < 0.001	6.4 (3.7-11), < 0.001

^a^Logistic regression model

## Discussion

Stillbirth rate is a sensitive indicator for assessing health status of a population. At the country level, it largely reflects the quality of ante-partum and intra-partum care available to pregnant women. Individually, stillbirth is a tragedy for the women and her family [29].

### Stillbirth rate

The stillbirth rate in Chandigarh Union Territory (UT) was estimated to be 16/1000 births using capture-recapture method, which is better than the Indian estimate (23/1000 live birth) by Blencowe et al. *(2016)* in 2015 [5]. There is a wide range of variation in the stillbirth rate in various states and union territories of India (12.5 to 26.48/1000 births). Similar variations have been observed in the infant mortality rate also [6–11]. Some of these variations may also reflect differences in methodologies that were adopted for gathering information on stillbirths [6–11]. Generalisability of our study is limited. Chandigarh is located in northern India which has better per capita income than rest of the county. This city has better intra-partum care facilities; and most of the women (91.6%) deliver in health institutions [15]. In Chandigarh, due to less distance and good road connectivity, women have better access to the health facilities. *Janani Shishu Suraksha Karyakaram* (Safe Maternity and Newborn Care Program) has been implemented in Chandigarh also for improving the intra-partum care [30].

### Causes of stillbirths

In our study antepartum stillbirths were found to be nearly 68%, whereas the intra-partum stillbirths were 32%. Lawn et al. (2011) have estimated intra-partum stillbirths to be 39% in middle-income countries [4]. There are nearly 35 classification systems for tabulating causes of stillbirths that have been published in past 50 years, and 15 among them were published in the past 15 years [3, 4]. The simplest categorization is based on time of stillbirth (ante-partum and intra-partum). This classification system is feasible even in the home births and it is also relevant programmatically for public health action [4]. Hence, we adopted this system to tabulate the causes after International Classification of Diseases (ICD) coding (Table 1). In order to move towards the single digit stillbirth rate target, in addition to intra-partum care, antepartum care will also plays an important role, as complications during the antepartum period are associated with the poor outcome of the pregnancy. Recently Government of India Ministry of Health and Family Welfare has formulated new guidelines for management of diabetes, hypothyroidism, calcium & iron supplementation and deworming during pregnancy under the *Janani Suraksha Yojna* (Maternity Security Program). A once-a-month fixed-day antenatal check-up campaign has also been launched (Pradhan Mantri Surakshit Matritva Abhiyan – Prime Minister Secure Motherhood Campaign) [31]. These initiatives can have impact major causes of stillbirth reported in our study (infections, medical conditions, growth retardation, induced labour etc.).

### Risk factors of stillbirths

Two key preventable or manageable findings in our study were congenital malformations and maternal hypertension. Significant association of stillbirth with maternal hypertension was also noticed in many studies including a systematic review [32]. Association of congenital malformations with stillbirths has been noticed in many studies in the developed countries and also in developing countries [3, 4]. Recently government of India has initiated measles and rubella vaccination campaign for all the children between 9 months to 15 years, which will prevent the congenital malformation due to rubella [33]. More emphasis should be given on folic acid supplementation to the mothers in the community who are planning to conceive so as to prevent neural tube defects [34]. Like other studies, our study also reported association of stillbirth with foetal growth restriction [35–37].

Another finding in our study is higher risk of stillbirth in vaginal deliveries. This association has been noticed in many other studies also [37, 38]. Women with stillbirth usually deliver vaginally unless otherwise there is an absolute indication for caesarean section [39]. Once the stillbirth is diagnosed there is higher chance to go for inducing labour to avoid complications [40]. Association of preterm birth with.stillbirth also noticed in our study like other studies [35–37].

Among the socio-demographic factors, socio-economic status, caste, and religion and were not significantly associated with stillbirth in this study. This may be due to the fact that controls were selected from the same community where cases resided (neighbourhood control). This strategy has resulted in matching for the socioeconomic status as people with similar socio-economic status generally live in the same neighbourhood. Interestingly, those who were having smaller household size had higher risk of stillbirth. Negative impact of smaller household size (nuclear families) on stillbirth observed in this study could be due to lower family support for managing pregnancy and child birth compared to those who have larger household size (joint families). Some studies have reported better social capital leads to desirable outcome of pregnancy [41–44].

### Study limitations

One of the limitations of retrospective population-based studies, such as ours, is the difficulty of classifying stillbirth into antepartum and intrapartum due to inadequately recorded data. However, since this classification has health policy and programme related implication, limited hospital records were supplemented with verbal autopsy interviews to classify stillbirths as antepartum and intrapartum in this study. One of the studies has noted that recall method used in verbal autopsy can lead to misclassification [4, 45]. Validation of verbal autopsy questions in classifying stillbirth as antepartum or intrapartum needs considered in future studies. Another limitation in this study was the use of estimated live births for calculating stillbirth rate. We consider use of crude birth rate from a sample survey and estimation of population from the Census has provided a reasonable estimate of the livebirths in Chandigarh UT. Out of the 301 stillbirths, we could trace only 120 due to wrong address provided to the hospitals and due to shifting of some women to other cities. This has led to reduction in sample size, and some of the risk factor estimates having very wide confidence intervals are difficult to interpret. Lastly, some of the risk factors are based on responses to verbal autopsy questions rather than hospital diagnosis.

In general, it is very difficult to differentiate between the ‘causes’ and the ‘risk factors’. For example, whether medical conditions such as hypertension and congenital malformations should be considered as causes or risk factors. We have included in the risk factor analysis only those variables which had not been judged to be the causes. However, categorization of variables into causes or risk factor would not matter while selecting interventions for prevention of stillbirths.

### Study strengths

Major strength of the study was that this is a population-based study. The capture and re-capture analysis showed that identification of stillbirths was appropriate. It has paved the way for health managers and public health experts for estimating stillbirth rate and causes/ risk factors of stillbirth to move forward in planning appropriate evidence-based strategies for reducing the stillbirth.

## Conclusions

The stillbirth rate in Chandigarh UT of India was 16/1000 birth during year 2013–14. Antepartum stillbirth were more common (68%) than the intra-partum stillbirths (32%). Major medical causes and risk factors were infections, maternal hypertension and congenital malformation which are amenable to health interventions. Smaller family size was found to have higher risk of stillbirth which needs further exploration. We recommend better ante-natal and intra-natal care can achieve the goal of single digit status of stillbirth rate by 2025.

## Additional file

Additional file 1: Table - Association of maternal and foetal causes of stillbirths. This cross tabulation shows the association between maternal and foetal causes of stillbirth. (DOCX 38 kb)

## Abbreviations

ANM: Auxiliary nurse midwife
aOR: Adjusted odds ratios
CI: Confidence interval
UT: Union Territory
