# Supplementary material for: Level, causes, and risk factors of stillbirth: a population-based case control study from Chandigarh, India
Source: BMC Pregnancy Childbirth. 2017 Nov 13;17:371. doi: 10.1186/s12884-017-1557-4 (PMC5684767; doi:10.1186/s12884-017-1557-4)
Supplement: Additional file 1: — Table - Association of maternal and foetal causes of stillbirths. This cross tabulation shows the association between maternal and foetal causes of stillbirth. (DOCX 38 kb) [file 12884_2017_1557_MOESM1_ESM.docx]

**Table: Association of maternal and foetal causes of stillbirths**

| **Maternal cause** | **Foetal cause** | | | | | Total |
| --- | --- | --- | --- | --- | --- | --- |
|  | Congenital anomaly | Infection or chorio-amnionitis | Foetal growth restriction/ placental insufficiency | Other specific foetal conditions | No conditions identified |  |
| Abnormal labour or uterine rupture | 2 | 0 | 2 | 0 | 5 | 9 |
| Maternal Hypertension | 3 | 0 | 14 | 2 | 14 | 33 |
| Maternal infection | 0 | 1 | 0 | 0 | 0 | 1 |
| Chorio-amnionitis | 3 | 15 | 4 | 1 | 2 | 25 |
| Maternal Diabetes | 0 | 0 | 1 | 0 | 2 | 3 |
| Antepartum haemorrhage | 0 | 0 | 1 | 0 | 5 | 6 |
| Maternal Pre-existing conditions | 0 | 0 | 1 | 0 | 1 | 2 |
| Spontaneous preterm labour | 4 | 0 | 2 | 1 | 9 | 16 |
| Other maternal specific conditions | 0 | 0 | 2 | 0 | 3 | 5 |
| No maternal conditions identified | 22 | 1 | 9 | 3 | 46 | 81 |
| Total | 34 | 17 | 36 | 7 | 87 | 181 |
